# Rare Case of Collecting Duct Carcinoma With Complete Response to Nivolumab

**DOI:** 10.1155/crom/9619945

**Published:** 2025-10-28

**Authors:** Muhammed Hajmusa, Gi Eun Kim, Mohammed Ussama Al Homsi, Ahmed Abdalhadi

**Affiliations:** ^1^Medical Oncology, National Center for Cancer Care and Research (NCCCR)–Hamad medical Corporation (HMC), Doha, Qatar; ^2^Internal Medicine, Hamad Medical Corporation (HMC), Doha, Qatar

## Abstract

Collecting duct carcinoma (CDC) is a rare, aggressive subtype of renal cell carcinoma originating in the renal medulla. We report a unique case of metastatic CDC in a patient with prior breast ductal carcinoma in situ. Genomic profiling revealed a homozygous deletion of CDKN2A (encoding p16). After progression on systemic therapies, the patient received stereotactic body radiotherapy (SBRT) to metastatic lesions concurrently with nivolumab (anti-PD-1). This regimen achieved a rapid complete radiographic remission of all lesions. We present a 63-year-old Sudanese woman with metastatic CDC who achieved a complete remission of over 5 years following nivolumab therapy. The patient initially presented with right flank pain and hematuria. Imaging revealed an exophytic renal mass, and she underwent radical nephrectomy in June 2019. Pathology confirmed high-grade CDC (pT3aN1) with clear margins; immunohistochemistry was notable for positive vimentin, PAX8, CK19, and patchy AMACR, with loss of CDKN2A. Postoperative PET/CT was clear, but by October 2019, three intra-abdominal metastases were seen (liver and retroperitoneum). She was first treated with palbociclib and letrozole, but progression occurred after 3 months. Given reports of immunotherapy efficacy in CDC, she began nivolumab in May 2020. Imaging in October 2020 showed marked tumor regression, sustained on repeat scans. In September 2021, isolated para-aortic lymph node recurrence was treated with stereotactic radiation (20–25 Gy/5 fractions) while continuing nivolumab. Subsequent PET/CT scans (Feb 2022, Feb 2023, June 2023, March 2024, and January 2025) demonstrated continued complete metabolic response. This combined modality approach achieved an unprecedented durable response, underscoring that personalized multimodal therapy—linking radiotherapy, immunotherapy and targeted cell-cycle inhibition—can yield long-term control in CDC. For a disease as lethal as CDC, this outcome demonstrates that genomically informed therapy can achieve extraordinary benefit.

## 1. Introduction

Collecting duct carcinoma (CDC) is a rare form of renal cell carcinoma (RCC), accounting for <1% of kidney tumors and typically arises from the renal medulla. It carries a poor prognosis due to aggressive behavior; up to 40% of patients have metastatic disease at diagnosis. In large CDC series, the median age is ~58 years, and most patients are symptomatic (e.g., flank pain and hematuria) [1]. Genomic studies frequently identify alterations in tumor suppressor genes (e.g., NF2, SETD2, SMARCB1, and CDKN2A) [2]. Traditional treatment for metastatic CDC has been platinum-based chemotherapy (gemcitabine + cisplatin/carboplatin), yielding modest responses (response rates ~26% and median survival ~7 months) [3]. There is no standard second-line therapy, and novel approaches are needed. Nivolumab is a monoclonal antibody against programmed death-1 (PD-1) that blocks PD-1/PD-L1 interaction, thereby restoring T-cell activity [4]. Immune checkpoint inhibitors like nivolumab have shown efficacy in various RCC subtypes and urothelial cancers.

## 2. Case Presentation

A 63-year-old Sudanese woman (married, with children) had a history of migraines, degenerative spinal disease, and prior right breast ductal carcinoma in situ (ER-positive, PR-negative, and HER2-negative) treated by modified radical mastectomy in 2021. In early 2019, she developed right flank pain and intermittent gross hematuria over several months. Workup by imaging revealed an exophytic solid renal mass on the right. She underwent radical nephrectomy in June 2019. Pathology confirmed high-grade CDC (pT3aN1, one of 13 lymph nodes positive) with clear margins. Immunohistochemical staining of the tumor showed vimentin(+), PAX8(+), CK19(+), INI1 retained, focal AMACR positivity, with negative PAX2, CK7, GATA3, OCT3/4, and CD10. Notably, cytogenetic testing revealed CDKN2A (p16) loss.

A PET/CT in August 2019 was negative for residual disease. However, by October 2019, repeat PET/CT showed three small metastatic lesions: a 1.5-cm nodule on the surface of liver Segment 6, and two subcentimeter nodules in the right retroperitoneum (near duodenum and para-aortic region). In November 2019, she began systemic therapy with palbociclib (a CDK4/6 inhibitor) plus letrozole. After 3 months, PET/CT demonstrated progression of all known lesions (no new sites). Given reports of CDC responding to immunotherapy, the decision was made to start nivolumab in May 2020.

**Figure 1:**
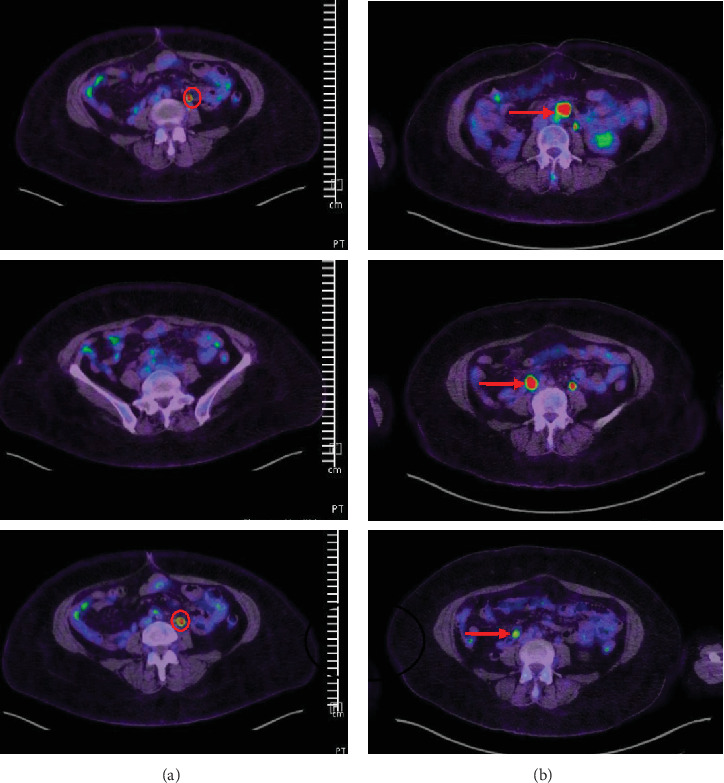
FDG PET scans in January 2025 (a) compared to October 2019 and April 2020 (b): The red arrows indicate metabolic activity in the right common iliac lymph nodes and retroperitoneal lymph nodes. The red circle denotes physiological uptake and excretion in the left ureter. The FDG PET scan from January 2025 (a) demonstrates sustained resolution of the retroperitoneal and right common iliac lymph nodes.

After four cycles of nivolumab, the October 2020 PET/CT showed marked reduction in metabolic activity of all lesions. By March 2021 (after 10 cycles), imaging showed near-complete metabolic response. In September 2021, however, PET/CT revealed metabolic recurrence in a para-aortic lymph node. She underwent stereotactic body radiotherapy (SBRT) to that node (20–25 Gy in five fractions) in October 2021, and nivolumab was continued. A follow-up PET/CT in February 2022 showed resolution of the retroperitoneal node. Subsequent PET/CTs in February 2023 and June 2023 confirmed sustained complete metabolic remission with no new lesions (Figure 1). The patient tolerated nivolumab well, with only mild fatigue, and continues on therapy with excellent quality of life.

## 3. Discussion

CDC is a rare and aggressive non-clear-cell RCC subtype. Due to its rarity, most data derive from retrospective analyses and case series [1, 3]. Median survival is typically under 1 year with conventional therapies. In recent years, checkpoint inhibitors have become standard in clear-cell RCC, but evidence in CDC is limited. To date, only a few case reports (seven prior cases) document nivolumab use in metastatic CDC [5–9]. Five of those cases presented with metastatic disease initially, while three (including ours) had localized disease at diagnosis but later developed metastases [6–8]. Treatment approaches varied: most underwent nephrectomy when resectable, and a minority received ipilimumab plus nivolumab combinations [5, 9].

Reported outcomes have ranged from progressive disease to complete remission. Three cases achieved partial responses (duration 2.5–6 months) and two had prolonged stable disease [6–8]. Only two prior reports noted complete responses, lasting 6 months to 3 years [5, 9]. Our patient's complete remission (≥ 3 years to last follow-up) is notable. Some authors noted high PD-L1 expression in CDC tumors responding to nivolumab [6–9], suggesting a potential biomarker, although PD-L1 was not tested in our patient. The CheckMate 214 trial established the efficacy of nivolumab+ipilimumab in metastatic RCC [4], and four of the reported CDC cases used this combination [5, 9].

An additional factor in our case is the use of radiotherapy. None of the prior case reports described combining immunotherapy with radiation. We administered SBRT to a recurrent node while continuing nivolumab. Radiation therapy, including SBRT, can enhance the immune response to immunotherapy (i.e., nivolumab) by causing immunogenic cell death and the release of tumor-associated antigens. This brings about activation of the cGAS–STING–interferon pathway, augmentation of the function of dendritic cells, as well as effective priming of T-cells. The resultant transition from an immune-cold to an immune-active tumor environment can optimize T-cell infiltration and augment the PD-1 blockade response [10].

The patient's sustained response supports the concept of a synergistic effect of radiation and immunotherapy. A retrospective study in CDC suggested a survival benefit for multimodal therapy (surgery + radiation + chemotherapy) over chemotherapy alone [11]. Our outcome aligns with that evidence. CDC-specific clinical trials are scarce. One small pilot study used nivolumab after cabozantinib in refractory CDC (*n* = 5), with median survival under 20 months [12].

In summary, this case is different from the previous reports by its integration of multiple modalities based on tumor biology. The concomitant use of SBRT with nivolumab, preceded by genomic-guided palbociclib/letrozole therapy, resulted in an extraordinarily durable remission that significantly exceeds previous CDC outcomes. One of the limitations of this case, PD-L1 status was not assessed in this patient nor the tumor mutation burden (TMB). The case highlights that even a deadly malignancy like CDC can be treated long-term when therapy is customized to the molecular and clinical setting. Although this is a single example, it provides proof of concept that combining immune checkpoint blockade, targeted radiation, and cell cycle inhibition can drastically change CDC's natural history. Further exploration of such tailored regimens in the CDC is necessary to establish if equally beneficial outcomes may be achieved in other patients.

## 4. Conclusion

We report a case of metastatic CDC with an exceptional response to nivolumab. This patient achieved a complete radiographic remission lasting at least 5 years, the longest reported to date for nivolumab-treated CDC, and the first noted with adjunctive radiotherapy. This case contributes to the accumulating evidence that immune checkpoint inhibitors can be effective in CDC. Our findings support considering nivolumab in metastatic CDC, particularly as part of a multimodal strategy, and highlight the pressing necessity for clinical trials in this uncommon tumor subtype.

## Data Availability

All data generated or analyzed during this case report is included in this published article.
